# Nutzerzentrierung in der öffentlichen Verwaltung: Welche Potenziale bietet Design Thinking?

**DOI:** 10.1365/s35764-022-00442-2

**Published:** 2022-12-05

**Authors:** Jonas Ruhe, Michael Neumann, Annette Heberlein

**Affiliations:** 1 Niedersächsische Landesbehörde für Straßenbau und Verkehr, Göttinger Chaussee 76A, 30453 Hannover, Deutschland; 2 Fakultät IV, Abt. Wirtschaftsinformatik, Hochschule Hannover, Ricklinger Stadtweg 120, 30459 Hannover, Deutschland

Die gesetzlich vorgesehene Bereitstellung von Digitalisierungsangeboten stellt öffentliche Verwaltungen vor steigende Herausforderungen. Aufgrund der Heterogenität der Nutzerinnen und Nutzer ist es für öffentliche Verwaltungen häufig problematisch, klare Anforderungen zu erheben und zu erfüllen. Hinzukommen strukturelle und organisatorische Gegebenheiten wie beispielsweise ausgeprägte Entscheidungshierarchien, die eine nutzerzentrierte Vorgehensweise erschweren können. Darüber hinaus sieht sich die öffentliche Verwaltung zunehmend mit komplexer werdenden Problemen konfrontiert. Es stellt sich daher die Frage, wie in der öffentlichen Verwaltung ein moderner Ansatz zur Nutzerzentrierung und Problemlösung eingesetzt werden kann. Dieser Artikel präsentiert die Ergebnisse einer Einzelfallstudie bei der Niedersächsischen Landesbehörde für Straßenbau und Verkehr (NLStBV). Wir haben mit einer Fokusgruppe einen Design-Thinking-Workshop durchgeführt, um Potenziale und Anwendungsmöglichkeiten des Ansatzes in der öffentlichen Verwaltung zu identifizieren. Auf Basis einer SWOT-Analyse haben wir die Ergebnisse untersucht und geben vier konkrete Handlungsempfehlungen für die Einführung sowie Nutzung von Design Thinking.

## Einleitung

Disruptive Veränderungen wie die digitale Transformation durchziehen nahezu alle Bereiche gesellschaftlichen Zusammenlebens. Durch die Verzahnung von Politik und Gesellschaft kann sich auch die öffentliche Verwaltung in Deutschland diesen Veränderungen nicht entziehen, was angemessene Reaktionen erfordert [1]. Die COVID-19-Pandemie zeigt deutlich, dass eine nach Rechtsstaatlichkeit, Hierarchie und Schriftlichkeit strebende öffentliche Verwaltung in solchen Situationen an ihre Grenzen kommt [2]. So blieben beispielsweise die Bürgerämter geschlossen und die Beschäftigten arbeiteten von einem Tag auf den anderen aus dem Homeoffice. Klassische Lösungsmuster wie das Aufstellen und Verfolgen von Plänen über einen Zeitraum von mehreren Jahren reichen nicht mehr aus, um nachhaltig und effizient zu agieren. Vielmehr gilt, auf sich ändernde Rahmenbedingungen schnell zu reagieren und Innovationen einzuführen. Sowohl privatwirtschaftlich organisierte Unternehmen [3] als auch öffentliche Verwaltungen [4] reagieren auf diese Entwicklung eines zunehmend volatilen, unsicheren und komplexen Umfeldes mit dem Einsatz agiler Methoden.

Darüber hinaus steigen die Anforderungen der Nutzerinnen und Nutzer an digitale Verwaltungsangebote. Mit dem Onlinezugangsgesetz (OZG) wurde die rechtliche Grundlage geschaffen, Verwaltungsdienstleistungen des Bundes, der Länder und der Kommunen bis Ende 2022 digital anzubieten. Im Rahmen der Umsetzung wurde vom IT-Planungsrat beschlossen, besonderen Wert auf die Nutzerfreundlichkeit der Angebote zu legen [5]. Der eGovernment Monitor zeigt jedoch, dass lediglich 47 % der Nutzerinnen und Nutzer mit den bislang angebotenen digitalen Leistungen zufrieden sind [6]. Dieser vergleichsweise niedrige Wert ist unter anderem durch die heterogenen Zielgruppen bei den Bürgerinnen und Bürgern begründet. Auch verwaltungsintern steigen die Anforderungen der Mitarbeiterinnen und Mitarbeiter an die Nutzung von Software, Fachverfahren und die IT-Umgebung [7]. Der Einsatz agiler Methoden zur Steigerung der Nutzerzentrierung ist bekannt. Looks et al. zeigen in ihrer Studie zu Agilität und Nutzerzentrierung in der öffentlichen Verwaltung, dass aufgrund der heterogenen Zielgruppen die Nutzerzentrierung eine bedeutende Facette in Bezug auf die digitale Transformation der öffentlichen Verwaltung darstellt [8]. Die Autoren formulieren Handlungsempfehlungen für die agile Transformation und Verbesserung der Nutzerzentrierung in der öffentlichen Verwaltung auf Basis von sechs Dimensionen: kommunikativ, änderungsaffin, produktgetrieben, teamzentriert, iterativ und verbesserungsorientiert.

Ein verbreiteter Ansatz, auf sich ändernde Rahmenbedingungen reagieren und Innovationen entwickeln zu können und gleichzeitig auf Wünsche sowie Bedürfnisse der Nutzergruppe einzugehen, ist Design Thinking (DT) [9]. Bei der Anwendung von DT werden insgesamt sieben Phasen iterativ durchlaufen, wobei stets das Ziel verfolgt wird, die zukünftigen Nutzerinnen und Nutzer mit ihren Bedürfnissen zu verstehen und Innovationspotenziale zu eruieren [10]. Der Ansatz eignet sich, um Probleme zu lösen, die einem komplexen Kontext zuzuordnen sind. Der kombinierte Einsatz von DT und agilen Methoden wird in diversen Studien untersucht. Pereira und Russo zeigen in ihrer systematischen Literaturrecherche, dass die Integration von DT in agile Methoden zu konkreten Vorteilen führen kann [11]. Die Autoren beschreiben, dass positive Auswirkungen auf die Kommunikation und Kollaboration zwischen agil operierenden Teams und Nutzern bzw. Kunden vorhanden sind. Dadurch steigt die Qualität der entwickelten Software. Zudem ist bekannt, dass DT in der öffentlichen Verwaltung und der Politik verwendet wird, um die sich aus der Nutzerzentrierung ergebenden Potenziale aufzugreifen und den aktuellen Herausforderungen adäquater zu begegnen [12, 13].

Ziel dieses Artikels ist es, die Potenziale von DT im Kontext der öffentlichen Verwaltung in Deutschland zu untersuchen, um Handlungsoptionen aufzuzeigen und die Entwicklung nutzerzentrierter Lösungen voranzutreiben. Dazu wurde in der Niedersächsischen Landesbehörde für Straßenbau und Verkehr (NLStBV) eine Fallstudie durchgeführt. Im Rahmen eines Design-Thinking-Workshops wurde der Ansatz in der Landesbehörde mit einer Fokusgruppe erprobt und evaluiert. Um die Potenziale des Ansatzes zu untersuchen, erfolge eine SWOT-Analyse. Auf Basis der skizzierten Ziele haben wir folgende Forschungsfragen (FF) definiert:

- *FF1:* Wie kann Design Thinking in der öffentlichen Verwaltung verwendet werden?
- *FF2:* Welche Potenziale bietet Design Thinking in Hinblick auf die Nutzerzentrierung im Kontext der öffentlichen Verwaltung?

Der Artikel ist wie folgt strukturiert: Im folgenden Kapitel beschreiben wir die zugrunde liegende Forschungsmethode. Anschließend werden die Ergebnisse der einzelnen Dimensionen der SWOT-Matrix vorgestellt. Aufbauend auf den Ergebnissen folgt ein Aufzeigen von Handlungsempfehlungen, bevor der Artikel mit einer Zusammenfassung und dem Ausblick schließt.

## Forschungsdesign

**Figure 1:**
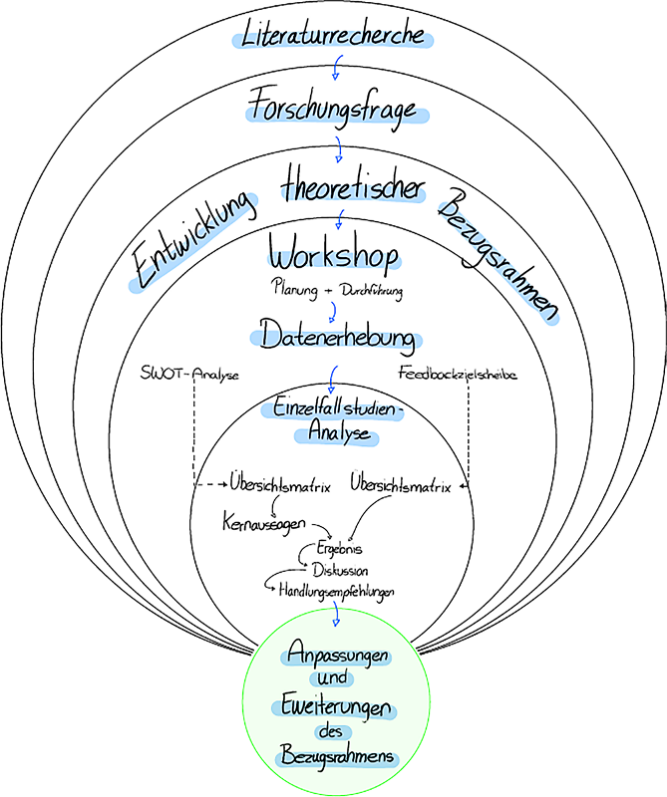

Die zugrunde liegende Studie haben wir als Einzelfallstudie nach der Guideline von Runeson und Hoest [14] konzipiert. Das Forschungsdesign präsentieren wir in Abb. 1. Auf Basis einer Literaturrecherche wurden die Forschungslücken identifiziert und konkrete Forschungsfragen abgeleitet. Um die Potenziale von DT im Kontext der öffentlichen Verwaltung zielgerichtet untersuchen zu können, haben wir einen Workshop vorbereitet, den wir mit einer Fokusgruppe durchgeführt haben. In den folgenden Unterabschnitten wird zunächst das Fallstudiendesign vorgestellt. Darauf aufbauend wird der zur Datenerhebung gewählte Ansatz erläutert, der auf Durchführung des Workshops mit einer Fokusgruppe basiert, ehe dargelegt wird, wie die Daten der Studie extrahiert und analysiert wurden.

Um zu prüfen, welches Cluster für das EC-Team relevant ist, wird eine Mehrpunktabfrage durchgeführt. Dadurch wird deutlich, dass insbesondere der Cluster Gesundheit im Arbeitskontext eine wichtige Rolle spielt. Infolgedessen wird ein Konzept für gesundheitsfördernde Maßnahmen im Rahmen des mobilen Arbeitens entwickelt, welches in Abschnitt *Fallstudie und Fokusgruppe* vorgestellt wird, um auf dieser Basis anschließend die Datenerhebung durchzuführen.

### Fallstudie und Fokusgruppe

Im Rahmen dieser Forschungsarbeit wird DT im Kontext der öffentlichen Verwaltung untersucht. Eine ausschließlich quantitative Forschungsmethode ist in Fällen, in denen Menschen miteinander in Interaktion treten, häufig nur bedingt aussagekräftig [14]. Der Kontext dieser Fallstudie ist die NLStBV. Für die Datenerhebung nutzen wir die Methode der Fokusgruppendiskussion [15]. Die Fokusgruppe setzt sich zusammen aus acht Mitarbeiterinnen und Mitarbeitern der NLStBV aus den Bereichen Informations- und Kommunikationstechnik, Changemanagement, Organisation, Planung und Umweltmanagement sowie Geoinformation. Die Mitarbeiterinnen und Mitarbeiter sind in Führungspositionen oder als Fachkräfte tätig. Die Datenerhebung in der Fokusgruppe haben wir auf Basis des Design-Thinking-Workshops durchgeführt. Der Workshop wurde nicht in Bild oder Ton aufgezeichnet. Neben einem handschriftlichen Protokoll diente insbesondere das virtuelle Whiteboard als Datenerhebungsinstrument.

### Datenextraktion und -analyse

Die Datenextraktion haben wir auf Basis des virtuellen Miro-Boards und schriftlicher Notizen mithilfe von Microsoft Excel durchgeführt. Die Tabelle haben wir auf Basis der SWOT-Dimensionen strukturiert. Die Daten des Miro-Boards wurden zunächst im Original überführt. Die Analyse der Daten erfolgte in einem zweistufigen Abstraktionsverfahren. Im ersten Schritt haben wir gleichartige Aussagen aus dem Workshop codiert und entsprechend kategorisiert. Die Kategorisierung diente dann als Basis zur Ableitung von Kernaussagen.

## Ergebnisse

In diesem Abschnitt werden die analysierten Ergebnisse des mit der Fokusgruppe durchgeführten Workshops bezüglich der Dimensionen Stärken und Schwächen sowie Chancen und Risiken im Kontext der öffentlichen Verwaltung vorgestellt.

### Stärken

Durch den Einsatz verschiedener, oftmals kreativer und interaktiver Methoden innerhalb der durchlaufenen Phasen wird der Ansatz als motivierend wahrgenommen. Die Arbeit in einem interdisziplinären Team sowie das Nichtvorhandensein von Hierarchien innerhalb des Teams führen dazu, dass alle Beteiligten ihre Perspektive mit einfließen lassen können. Dies fördert wiederum die Motivation. Darüber hinaus entstehen durch die verschiedenen Perspektiven neue Ideen und damit Potenziale für Innovationen. Die vielschichtige Untersuchung des Problemraumes steht dabei im Vordergrund. Innerhalb des Teams wird ein gemeinsames Verständnis der zu lösenden Herausforderungen erreicht. Erst nach einer detaillierten Untersuchung werden Lösungen entworfen. Daher wird der Ansatz als ganzheitlich wahrgenommen. Zudem ist der Einstieg in die Anwendung der Methodik als niederschwellig anzusehen. Der Fokus der zu bearbeitenden Fragestellung kann sich mitunter verschieben. Der Einsatz von DT ermöglicht ein Anpassen der Fragen während der Durchführung. Der gesamte Prozess oder Teile davon können mehrfach durchlaufen werden, was gerade dann relevant wird, wenn unter den Beteiligten das Gefühl unklarer, fehlerhafter oder fehlender Informationen entsteht. Die verschiedenen Phasen geben die Struktur des Prozesses vor. Aus diesem Grund nehmen die Beteiligten den Ansatz als strukturierte Herangehensweise wahr. Der Ansatz ermöglicht ein Arbeiten, das sich von den bekannten Verwaltungsstrukturen löst und aus diesen „ausbricht“.

### Schwächen

In der Dimension der Schwächen wurde deutlich, dass der Einsatz von DT trotz des vergleichsweise niederschwellig anzusehenden Einstiegs Zeit und Übung benötigt. Viele der in den einzelnen Phasen eingesetzten Methoden waren den Teilnehmenden unbekannt und bedurften daher einer einführenden Erläuterung. Weiterhin wurde die Notwendigkeit eines klaren Zeitmanagements verdeutlicht, da andernfalls unbekannte Methoden zu viel Zeit in Anspruch nehmen. Resultierend aus dem Einsatz unbekannter Methoden in Kombination mit der fehlenden Übung, kann es unter Umständen dazu kommen, dass der Fokus verloren geht. Darüber hinaus können die Arbeit in einem interdisziplinären Team und die Betrachtung der Problemstellung aus unterschiedlichen Blickwinkeln dazu führen, dass der Fokus aus dem Blick gerät. Mit zunehmender Übung im Einsatz der Methoden verringert sich der notwendige Zeitbedarf und die Qualität der Ergebnisse steigt. Um dies zu erreichen, erfordert DT eine grundsätzliche Offenheit für Neues. Eine positive Einstellung gegenüber der Abkehr von bekannten bürokratischen Verwaltungsstrukturen ist notwendig. Das Nichtvorhandensein hierarchischer Strukturen oder die Freiheiten, mit denen die Methodik eingesetzt werden kann, sind Beispiele für die unter dem Begriff der Bürokratie subsumierten Merkmale. Im Rahmen von DT wird eine Teamgröße von maximal neun Personen empfohlen. Ein weiterer als Schwäche genannter Aspekt ist daher die theoretische Begrenzung der Teamgröße. Zudem wurde der Einsatz von oftmals auf Brainstorming basierenden Methoden als Schwäche des Ansatzes angesehen.

### Chancen

Durch den Einsatz von DT besteht für die öffentliche Verwaltung die Chance, als attraktiver Arbeitgeber wahrgenommen zu werden. Der Einsatz moderner Vorgehensweisen kann dazu führen, dass potenzielle Arbeitnehmerinnen und Arbeitnehmer Interesse an einer Tätigkeit in der öffentlichen Verwaltung zeigen. Außerdem bietet der Ansatz die Möglichkeit, bei Fragestellungen angewendet zu werden, die in der Vergangenheit mit klassischen Vorgehensweisen nicht gelöst werden konnten. Das Betrachten der Fragestellung aus unterschiedlichen Blickwinkeln wird ebenfalls in der Dimension der Chancen aufgeführt. Allerdings verschiebt sich dabei der Kontext geringfügig. Der Austausch von Wissen, der durch die fachlichen Diskussionen angeregt wird, eröffnet den Teilnehmenden die Möglichkeit, auch nach Ende des Workshops ein Verständnis für die Arbeit anderer aufzubringen. Anhand der entstandenen Transparenz kann Wissen über die Grenzen der Organisationseinheiten hinweg geteilt werden. Durch die Arbeit auf einem (virtuellen) Whiteboard oder die Arbeit mit Zettel und Papier gemeinsam in einem Raum lässt sich das Vorgehen zudem ohne großen Mehraufwand sukzessive dokumentieren.

### Risiken

Mit der Dimension der Risiken im Rahmen der externen Analyse werden die Entwicklungen im Umfeld beschrieben. Die Rahmenbedingungen der öffentlichen Verwaltung können unter Umständen ein Hindernis bei der Umsetzbarkeit von DT darstellen. Insbesondere die Durchführung eines Design-Thinking-Workshops in einem Online-Format, in dem sensible Fragestellungen bearbeitet werden, könnte durch Datenschutzgesetze eingeschränkt werden. Rechtliche und organisatorische Hürden bei der Vorbereitung und Durchführung könnten dazu führen, dass der Ansatz in der öffentlichen Verwaltung nicht oder lediglich eingeschränkt Anwendung finden kann. Ein weiteres Risiko wird in den Folgen eines unreflektierten Einsatzes von DT gesehen. Ein solches Vorgehen ohne Beachtung des Kontextes kann dazu führen, dass die zu beantwortende Fragestellung aus dem Fokus gerät. Vielmehr sollte den Umfeldfaktoren ebenfalls Beachtung geschenkt und die zu lösende Frage in den Kontext eingeordnet werden. Entscheidend ist der Gesamtkontext, in dem der Ansatz angewendet werden soll. Bei Nichtbeachtung kann es unter Umständen dazu kommen, dass der angestrebte Erfolg nicht erzielt wird. Ein weiterer Aspekt ist eine möglicherweise negative Außenwirkung auf Mitarbeiterinnen und Mitarbeiter, die nicht Teil des DT-Teams sind. Im Rahmen von DT werden Techniken und Methoden eingesetzt, die oftmals unbekannt sind und die zugleich zu einem Loslösen von gewohnten Verwaltungsstrukturen führen. Die vorhandene Skepsis Neuem gegenüber kann sich zu einer grundsätzlich negativen Haltung in Bezug auf die Methodik entwickeln und zur Folge haben, dass es an Unterstützung mangelt.

## Handlungsempfehlungen

In diesem Abschnitt beschreiben wir vier konkrete Handlungsempfehlungen für den Einsatz von DT in der öffentlichen Verwaltung, die wir auf Basis der Ergebnisse unserer Studie erarbeitet haben.

### Vorschlag 1: Anwendung von Design Thinking

Die methodische Lösung komplexer Probleme stellt Organisationen vor erhebliche Herausforderungen, insbesondere da die Probleme in den vergangenen Jahren in ihrer Komplexität gestiegen sind. Dies wiederum kann auf die zunehmende Volatilität von Märkten und auch auf die COVID-19-Pandemie zurückgeführt werden, die wie ein Beschleuniger für diverse Transformationsvorhaben gewirkt hat. Hiervon ist die öffentliche Verwaltung, insbesondere durch ihre organisatorischen Strukturen und ausgeprägten Entscheidungshierarchien, ebenfalls betroffen. Es stellt sich daher die Frage, auf welche Weise methodische Ansätze wie DT zur Problemlösung konkret genutzt werden können und was es dabei zu beachten gilt. Aufgrund der Unkompliziertheit von DT ist das Einbinden verschiedener Stakeholder zur Lösung eines Problems ohne größeren Aufwand möglich. Darüber hinaus sieht DT vor, dass alle Beteiligten integriert werden und so die Motivation zur Interaktion sowie Kollaboration erhöht ist. Eine Herausforderung beim Lösen komplexer Problemen ist häufig die vielschichtige und detaillierte Analyse der zugrunde liegenden Schwierigkeiten. Auch hierfür ist DT gut geeignet, da für den Übergang zur jeweils nächsten Aktivität ein gemeinsames Verständnis über die Zwischenergebnisse vorhanden sein muss. Es kann also das unterschiedliche Fachwissen der Beteiligten einbezogen werden. Des Weiteren erlaubt DT aufgrund des iterativen Ansatzes ein Umsteuern oder Neujustieren auch während der Durchführung. Vor allem bei komplexen Problemstellungen kann es plausibel sein, den DT-Prozess oder einzelne Aktivitäten mehrfach zu durchlaufen, um dadurch die Qualität des Ergebnisses zu erhöhen. Darüber hinaus ist es üblich, die jeweiligen Aktivitäten mit Mikropraktiken (beispielsweise Brainstorming) durchzuführen oder methodenbasierte Ansätze (beispielsweise Ursache-Wirkung-Analysen oder Beobachtungen) zu nutzen. Wir weisen hierbei ausdrücklich darauf hin, dass für die Qualität der jeweiligen Problemlösung die Wahl einer Mikropraktik oder Methode von wesentlicher Bedeutung ist, und empfehlen daher, erfahrene Personen bei der Auswahl einzubeziehen. Durch den klar strukturierten Aufbau des DT ist also eine hohe Flexibilität bei der Durchführung gegeben. Diese kann es ermöglichen, bekannte Denk- und Verhaltensmuster abzulegen und losgelöst von den organisatorischen Strukturen in öffentlichen Verwaltungen Problemlösungen „neu zu denken“. Wichtig ist aus unserer Sicht, vorab ein Time-Boxing zu definieren und den Teilnehmenden bekannt zu geben. Andernfalls besteht das Risiko, den Fokus auf die jeweilige Zielsetzung bzw. Fragestellung der Aktivität zu verlieren und sich zu intensiv mit themenfremden Diskussionen aufzuhalten. Sollte jemand an den Workshops teilnehmen, der noch keine oder geringe Erfahrung mit DT gesammelt hat, empfehlen wir, das Time-Boxing etwas großzügiger zu gestalten, um den zeitlichen Druck abzumildern und potenziellen Rückfragen ausreichend Raum zu geben. Es sollte zudem berücksichtigt werden, dass auch die Anwendung von DT insbesondere unter Nutzung verschiedener Mikropraktiken oder Methoden Übung erfordert. Insbesondere die Diskussion der Problemstellung aus unterschiedlichen Perspektiven (beispielsweise Fachbereichen) kann dazu führen, dass die ursprüngliche Fragestellung nicht mehr im Mittelpunkt der Erörterung steht. Wir empfehlen daher, zumindest eine Person mit fundierten DT-Kenntnissen zur Moderation einzusetzen.

### Vorschlag 2: Optimierte Nutzerzentrierung mit Design Thinking

Wie oben beschrieben, ist eine zentrale Herausforderung in Software-Entwicklungsprojekten bei der öffentlichen Verwaltung die Heterogenität der Nutzerinnen und Nutzer. Die Frage nach einer potenziell plausiblen Nutzerzentrierung kann in diesem Kontext also komplex sein. Im Gegensatz zu unserem zweiten Vorschlag legen wir hier den Fokus auf die Herausforderung, wie wir die Nutzerzentrierung in Software-Entwicklungsprojekten optimieren können. Die Idee, eine Benutzerorientierung in Software-Entwicklungsprojekten zu gewährleisten, ist bekannt. Beim Einsatz agiler Methoden sind zudem diverse Praktiken und Techniken zur Optimierung der Qualität von Anforderungen weitverbreitet. Der Einsatz von DT kann hierbei insbesondere zu Beginn eines Projekts hilfreich sein. Durch den Ansatz, zunächst Visionen und Lösungspotenziale zu identifizieren sowie die Transparenz und Eindeutigkeit in den Zielsetzungen von Software-Entwicklungsprojekten zu verbessern, entstehen Klarheit und ein konsistentes Verständnis über die Stakeholder. Folglich fällt es leichter, die Nutzerinnen und Nutzer in das Projekt zu integrieren. Darauf aufbauend können in Kombination zielgerichtet bekannte Praktiken und Methoden aus der agilen Softwareentwicklung und der Nutzerzentrierung verwendet werden, um den Nutzen und die Akzeptanz des Softwareprodukts zu erhöhen. Aus unserer Sicht kann es hierbei von Vorteil sein, beide iterativen Ansätze, d. h. agile Methoden und DT, integrativ zu verwenden. Insbesondere bei agilen Methoden mit inkrementell ausgerichteter Bereitstellung neuer Funktionen eines Produktes erscheint dies ratsam. Ebenso empfiehlt es sich, Methoden von nutzerzentrierten Ansätzen zu verwenden, beispielsweise im Rahmen von Laboren vorzugehen und mithilfe von Prototyping qualitativ hochwertiger Mock-ups oder Click-Dummies die Nutzergruppen (oder deren Vertreter) frühzeitig in die Softwareentwicklung zu integrieren. Erforderlich hierfür ist die Erkenntnis, sich der permanent wechselnden Rahmenbedingungen und Anforderungen bewusst zu sein. Darüber hinaus weisen wir darauf hin, dass die Bereitschaft bei den Fachkräften und Leitungspersonen gegeben sein muss, experimentierfreudig zu agieren sowie eine entsprechende Fehlerkultur zu etablieren. Zudem ist hierbei das Risiko zu berücksichtigen, dass mehrere Transformationen gleichzeitig vonstattengehen werden. So werden zwar agile Methoden bereits teilweise in der öffentlichen Verwaltung einerseits angewendet, andererseits kann nicht davon ausgegangen werden, dass hohe Reifegrade bei der Verwendung dieser Methoden vorhanden sind.

### Vorschlag 3: Nachhaltigkeit von Design Thinking nutzen

Mit zunehmender Anzahl durchgeführter Workshops zu DT werden Fragen zur Dokumentation der Ergebnisse aufkommen. Hierbei ist zu berücksichtigen, dass bei der Nutzung von DT die systematische Dokumentation der jeweiligen Aktivitäten zu empfehlen ist. Nicht zuletzt aufgrund der Sicherstellung der empirischen Ergebnisse ist es ratsam, eine nachvollziehbare Struktur für die Dokumentation zu wählen, um im Zweifel in durchgeführten DT-Workshops noch einmal nach konkreten Aspekten suchen zu können. Wir raten zudem, die Ergebnisse möglichst transparent zur Verfügung zu stellen. So können die Teilnehmer auf Basis einer einheitlichen Struktur in ihren Fachbereichen von den Workshops berichten; auf diese Weise wird zugleich eine breitere Zielgruppe erreicht. Transparenz ist zudem Grundvoraussetzung für eine stetige Selbstoptimierung (Kaizen). Wenn wir bei der Anwendung von DT nachhaltig besser werden wollen, sind wir auf transparente Dokumentationen angewiesen. Auch wenn wir die Komplexität bewusst zur Transition von DT gering halten möchten, erscheint es plausibel, von Beginn an deutlich zu machen, dass wir auch unsere problem- oder projektbezogenen Design-Thinking-Workshops mit einem prozessorientierten DT-Ansatz optimieren können. Uns ist bewusst, dass hierfür weitere (agile) Praktiken wie Retrospektiven oder Lessons-Learned-Meetings zur Verfügung stehen. Dennoch kann es ratsam sein, auch aus einer organisatorischen Perspektive immer wieder selbst an Design-Thinking-Workshops mit dem Ziel teilzunehmen, das eigene Vorgehen zu hinterfragen und zu verbessern. Zu berücksichtigen ist in jedem Fall die Gegebenheit der jeweiligen öffentlichen Verwaltung. Es kann Restriktionen in Behörden oder Verwaltungen geben, die einen nachhaltigen Einsatz von DT, beispielsweise durch eine strukturierte Dokumentation und die transparente Bereitstellung der Ergebnisse, nicht ermöglichen. Insbesondere durch Datenschutzrichtlinien oder Geheimhaltungsvereinbarungen kann dies erschwert werden.

## Fazit

Dieser Artikel präsentiert die Ergebnisse unserer Einzelfallstudie in der Niedersächsischen Landesbehörde für Straßenbau und Verkehr (NLStBV), in der wir die Möglichkeit der Verwendung und die Potenziale des DT in der öffentlichen Verwaltung untersucht haben. Hierzu haben wir mit einer Fokusgruppe einen Workshop unter Anwendung von DT durchgeführt. Die gesammelten Daten haben wir mithilfe einer SWOT-Analyse systematisch analysiert und entsprechend der vier Dimensionen zugeordnet. Im Vergleich zu potenziellen Schwächen und Risiken kann festgehalten werden, dass mit dem Einsatz von DT in der öffentlichen Verwaltung überwiegend Chancen und Verbesserungspotenziale verbunden sind. Die Ergebnisse der Fallstudie zeigen, dass der Einsatz von DT in der öffentlichen Verwaltung von verschiedenen Stärken geprägt ist.

Darüber hinaus präsentieren wir vier konkrete Handlungsempfehlungen. Die erste Handlungsempfehlung thematisiert die Einführung von DT im Kontext der öffentlichen Verwaltung. Des Weiteren geben wir zwei Handlungsempfehlungen für den konkreten Einsatz von DT mit dem Ziel zur Lösung von Problemstellungen und zur Optimierung der Nutzerzentrierung in Software-Entwicklungsprojekten. Die vierte Handlungsempfehlung thematisiert die Nachhaltigkeit der gewonnenen Erfahrungen beispielsweise durch den Wissenstransfer in andere Bereiche der NLStBV.

Im Rahmen unserer Studie haben wir festgestellt, dass sowohl Ansätze und Methoden zur Nutzerzentrierung und Benutzerorientierung als auch agile Methoden und DT in der öffentlichen Verwaltung bereits eingesetzt werden. Dennoch existiert nach aktuellen Erhebungen ein Optimierungspotenzial insbesondere im Hinblick auf die Zufriedenheit der Nutzerinnen und Nutzer mit den Digitalisierungsangeboten der öffentlichen Verwaltungen. Es kann davon ausgegangen werden, dass die Einführung agiler Methoden und die Nutzerzentrierung in öffentlichen Verwaltungen erst am Anfang stehen und hier weiterer Forschungsbedarf vorhanden ist. Insbesondere zu der Frage, wie agile und hybride Methoden gemeinsam mit DT konkret verwendet werden können, sind detaillierte Erkenntnisse notwendig, um beispielsweise ein grundlegendes Verständnis darüber zu gewinnen, für welche Projektvorhaben sich diese Kombination eignet.

Disruptive Veränderungen üben einen Einfluss auf die Arbeit in der öffentlichen Verwaltung aus.

Hinzu kommt eine ausgeprägte Komplexität durch die vorhandene Heterogenität der Nutzer von digitalisierten Verwaltungsdienstleistungen.

Es stellt sich daher die Frage, welche Potenziale das Design Thinking in der öffentlichen Verwaltung bietet.

### Zusammenfassung

- Design Thinking ist ein systematischer Ansatz zur Entwicklung nutzerzentrierter Lösungen.
- Der Ansatz beruht auf dem iterativen Durchlaufen von insgesamt 7 verschiedenen Phasen, im Rahmen derer einzelne Methoden eingesetzt werden.
- Beispielhaft zu nennen sind hier das Brainstorming oder ähnlich gelagerte Kreativitäts- und Innovationstechniken.

### Handlungsempfehlungen

- Die Anwendung von Design Thinking erfordert die Integration der Stakeholder.
- Durch die kombinierte Nutzung von Design Thinking und agilen Methoden können Synergien im Sinne der optimierten Nutzerzentrierung geschaffen werden.
- Bei der Anwendung von Design Thinking ist Transparenz zu berücksichtigen, um eine stetige Verbesserung der vorhandenen Prozesse zu gewährleisten und so einen nachhaltigen Einsatz der Methode zu erreichen.
